# Role of Al in Na-ZSM-5 zeolite structure on catalyst stability in butene cracking reaction

**DOI:** 10.1038/s41598-020-70568-z

**Published:** 2020-08-12

**Authors:** Chanon Auepattana-aumrung, Victor Márquez, Sippakorn Wannakao, Bunjerd Jongsomjit, Joongjai Panpranot, Piyasan Praserthdam

**Affiliations:** 1 Center of Excellence On Catalysis and Catalytic Reaction Engineering, Department of Chemical Engineering, Faculty of Engineering, Chulalongkorn University, Bangkok, 10330 Thailand; 2 SCG Chemicals, Co., Ltd., 1 Siam Cement Road, Bangsue, Bangkok, 10800 Thailand

**Keywords:** Heterogeneous catalysis, Solid-state NMR

## Abstract

The Na-ZSM-5 catalysts (SiO_2_/Al_2_O_3_ molar ratio = 20, 35, and 50) were prepared by rapid crystallization method to investigate their performance in butene cracking reaction. The XRD, XRF, NH_3_-TPD, FT-IR, TPO, UV–Vis, and ^1^H, ^27^Al, ^29^Si MAS NMR techniques were used to identify the physical and chemical properties of Na-ZSM-5 catalysts. The silanol group (Si–OH) was the main acid site of Na-ZSM-5, and it was proposed to be the active site for the butene cracking reaction. The butene conversion and coke formation were associated with the abundance of silanol groups over the Na-ZSM-5 catalyst. The dealumination, resulting in the deformation of tetrahedral framework aluminum species was a key factor for Na-ZSM-5 catalyst deactivation, because of the Si–O–Al bond breaking and formation of Si–O–Si bond. The stability of the Si–O–Al bond was linked to the molar number of sodium since the Na atom interacts with the Si–O–Al bond to form Si–ONa–Al structure, which enhances the stability of the silanol group. Therefore, the Si–ONa–Al in zeolite framework was an essential structure to retain the catalyst stability during the reaction. The Na-ZSM-5 with the lowest SiO_2_/Al_2_O_3_ molar ratio showed the best performance in this study resulting the highest propylene yield and catalyst stability.

## Introduction

Currently, the catalytic cracking technology of C_4_ alkenes is one of the most attractive processes to fulfill the global propylene demand, since numerous C_4_ alkene products can be attained from traditional technologies such as fluid catalytic cracking (FCC)^1^. For the hydrocarbon cracking method, various types of catalysts have been developed, especially ZSM-5 zeolites-based because of their suitable physical and chemical properties^2,3^. However, the catalyst deactivation is one of the most crucial problems in the cracking of hydrocarbon reaction. Dealumination and coke formation are generally two main factors for the zeolite catalyst deactivation. The dealumination process, is an irreversible mechanism, regards the displacement of aluminum in tetrahedral coordination of zeolite framework, leading to the loss of Brönsted acid sites of the catalyst^4,5^. The Brönsted acid sites over the zeolite surface are active sites in various reactions, like hydrocarbon cracking reaction^6,7^. The coke deposition on catalyst, blocking the active sites for the reaction^8,9^ is the second cause for catalyst deactivation. This process is a reversible mechanism since the carbonaceous species can be burned off (catalyst regeneration) under the mild condition^10^. The coke formation mainly occurs at the strong Brönsted acid sites over the catalyst^11^, indicating that the number of coke species relates to the strong acid site density (Si–(OH)–Al) over the catalyst^12,13^. Previous research described the H-ZSM-5 catalyst deactivation due to coke formation during hydrocarbon cracking reaction, including coke location, coke species, and nature of coke deposition^14–16^. Na-ZSM-5 was found to be better than H-ZSM-5 for the 1-butene cracking reaction according to previous studies^17^. However, the catalyst deactivation of Na-ZSM-5 catalyst in this reaction has not been widely studied. Therefore, investigating the cause of the catalyst deactivation, especially in the absence of Si–(OH)–Al site^18^, is critical for proper understanding of the process.

One of the most powerful characterizations used to explore the zeolite properties, especially solid-acid zeolite catalyst, is Solid-state Nuclear Magnetic Resonance (NMR) spectroscopy^19,20^. The ^1^H MAS NMR method is also applied to identify the types of hydroxyl groups on solid catalysts, including Si–(OH)–Al, Si–OH, and Al–OH sites in zeolites^21^. The ^27^Al MAS NMR technique can provide the information on the different framework and extra-framework aluminum species in the zeolite, such as tetrahedral (Al^IV^), penta-coordinated (Al^V^), and octahedral (Al^VI^) aluminum species^20^. The structure of Al atoms nearby Si atoms are identified by the ^29^Si MAS NMR technique to investigate the binding structure of Si atoms like Si(4Si,0Al), Si(3Si,1Al), Si(2Si,2Al), Si(1Si,3Al), and Si(0Si,4Al) sites. Thus, Solid-state NMR spectroscopy is a powerful technique to properly characterize the solid-acid zeolite catalyst.

In this research, the catalyst deactivation phenomena over Na-ZSM-5 (with different SiO_2_/Al_2_O_3_ molar ratio) during the catalytic cracking reaction of butene were studied, especially the nature of coke formation and aluminum migration over Na-ZSM-5. The zeolite structure of synthesized Na-ZSM-5 catalyst, fresh and spent, was confirmed by XRD. The bulk sodium concentration of catalysts was characterized by XRF technique. The acid strength and OH groups stretching region of catalysts were identified by NH_3_-TPD and FT-IR techniques, respectively. The ^1^H MAS NMR method was employed to determine the acidic structure on Na-ZSM-5 catalyst, whereas the ^27^Al and ^29^Si MAS NMR spectroscopies were applied to describe the catalyst dealumination and the types of Al atoms surrounding Si atoms on the synthesized zeolite. The nature of coke species, and coke content over the catalyst were investigated by UV–Vis, and TPO techniques, respectively.

## Results

### Catalyst physical and chemical properties

The XRD pattern of synthesized Na-ZSM-5 catalysts, fresh and spent, are depicted in Supplement Figure S1. The fresh and spent Na-ZSM-5 catalysts presented the typical MFI structure. The sodium content on the bulk of catalyst was measured by XRF analysis and summarized in Supplement Table S1. The sodium content on the bulk of catalysts decreased with increasing the SiO_2_/Al_2_O_3_ molar ratio of Na-ZSM-5 catalyst.

**Figure 1:**
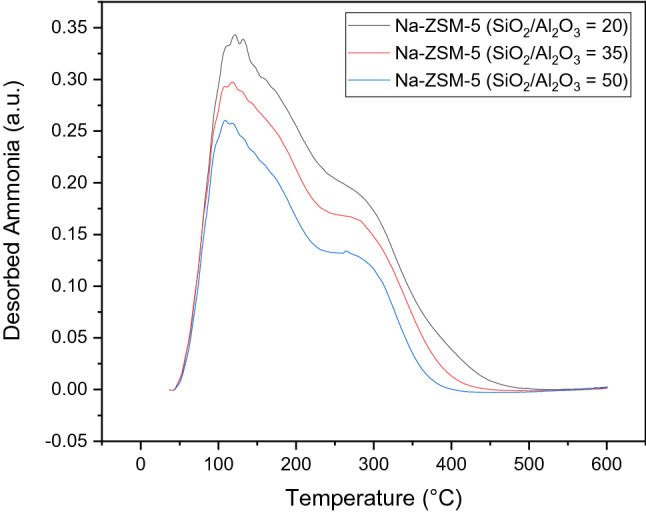
NH_3_-TPD profile of Na-ZSM-5 catalysts with different SiO_2_/Al_2_O_3_ molar ratios.

The acid strength and acidity number on Na-ZSM-5 catalysts with various SiO_2_/Al_2_O_3_ molar ratios are displayed in Fig. 1 and summarized in Supplement Table S2. Based on Fig. 1, NH_3_-TPD profiles on Na-ZSM-5 catalysts presented two main desorption peaks, related to weak and medium acid sites at about 120 °C and 270 °C, respectively. The number of total acid sites, weak and medium, increased when decreasing the SiO_2_/Al_2_O_3_ molar ratio, as detailed in Supplement Table S2. The OH stretching region of Na-ZSM-5 catalyst is detected by FT-IR as illustrated in Supplement Figure S2. The bands at ca. 3,490, 3,580, 3,685, and 3,745 cm^−1^ were assigned to silanol nests^22^, OH groups interacting with multivalent cations (negative charge compensation) in the zeolite framework^23^, internal silanol groups of hydroxyl nests^24^, and external silanols^25^, respectively. When increasing the SiO_2_/Al_2_O_3_ molar ratio of Na-ZSM-5, the band intensities related to silanol groups decreased as portrayed in Supplement Figure S2.

**Figure 2:**
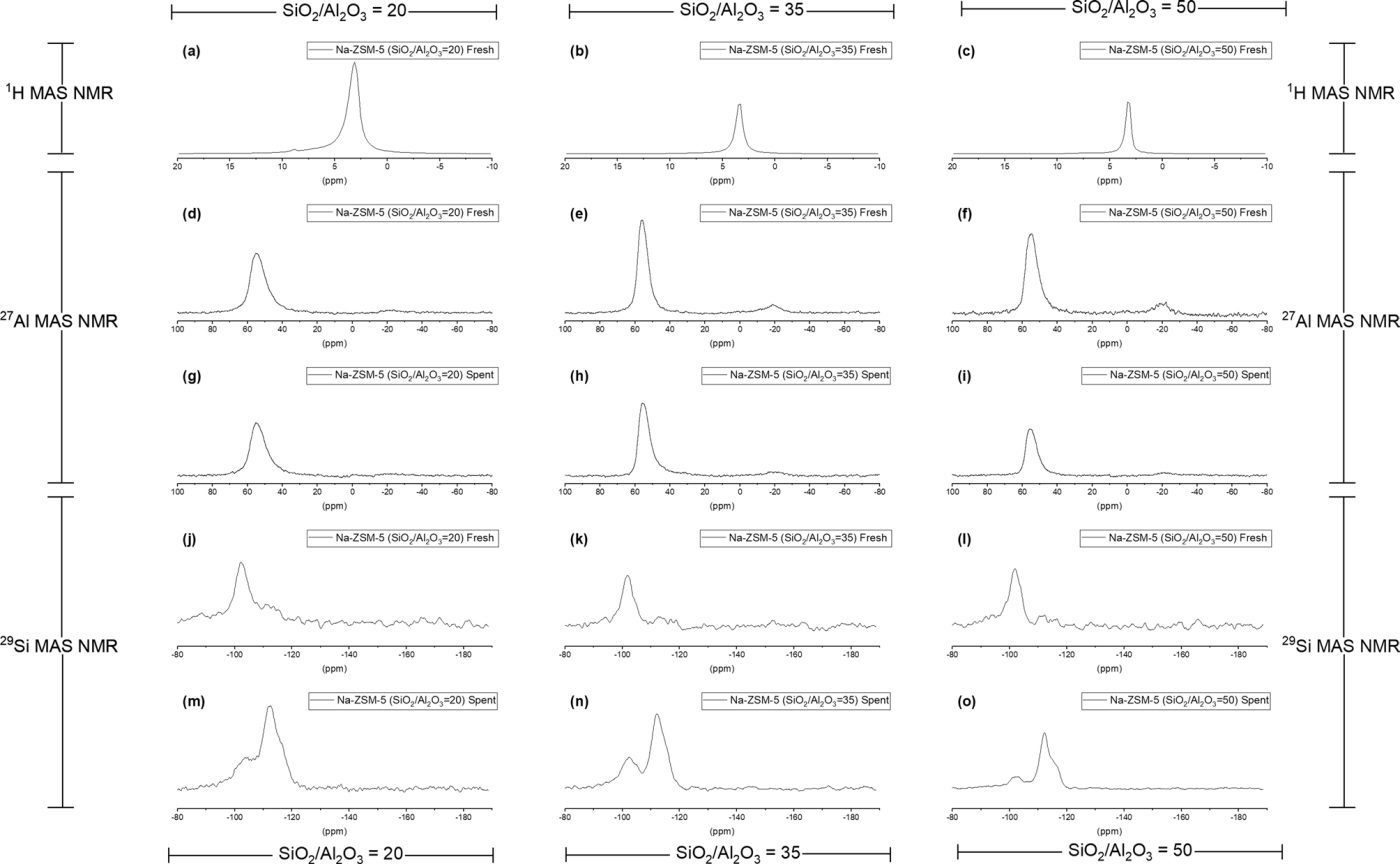
^1^H, ^27^Al, and ^29^Si MAS NMR of Na-ZSM-5 catalysts with different SiO_2_/Al_2_O_3_ molar ratios (**a**–**c**) ^1^H MAS NMR of fresh Na-ZSM-5 catalysts with the SiO_2_/Al_2_O_3_ molar ratios = 20, 35, and 50, respectively, (**d**–**f**) ^27^Al MAS NMR of fresh Na-ZSM-5 catalysts with the SiO_2_/Al_2_O_3_ molar ratios = 20, 35, and 50, respectively, (**g**–**i**) ^27^Al MAS NMR of spent Na-ZSM-5 catalysts with the SiO_2_/Al_2_O_3_ molar ratios = 20, 35, and 50, respectively, **j**–**l**^29^Si MAS NMR of fresh Na-ZSM-5 catalysts with the SiO_2_/Al_2_O_3_ molar ratios = 20, 35, and 50, respectively, and (**m**–**o**) ^29^Si MAS NMR of spent Na-ZSM-5 catalysts with the SiO_2_/Al_2_O_3_ molar ratios = 20 ,35, and 50, respectively.

^1^H MAS NMR spectra of fresh synthesized Na-ZSM-5 catalysts, displayed in Fig. 2a–c and summarized in Supplement Table S3, indicate the types of zeolite acidic structures. The main resonance signal between 3.2 and 3.4 ppm was attributed to the presence of Si–OH groups^26^. The signal of synthesized Na-ZSM-5 with SiO_2_/Al_2_O_3_ = 20, 35, and 50 were located at 3.2, 3.4, and 3.2 ppm, respectively. Based on Fig. 2a–c, no Si–(OH)–Al peak was detected over the synthesized Na-ZSM-5 catalysts. Therefore, Na-ZSM-5 could have only one type of Si–OH structure, which exhibited weaker acid strength than Si–(OH)–Al site (strong Brønsted acid site). The Si–OH ^1^H MAS NMR band intensity, as well as its abundance, decreased while increasing SiO_2_/Al_2_O_3_ molar ratio of synthesized Na-ZSM-5 as shown in Supplement Table S3.

^27^Al MAS NMR technique was used to identify the aluminum framework and extra-framework species in zeolite catalysts^20,27,28^. Figure 2d–i and Supplement Table S3 present the profile and density of the aluminum species in zeolite structure, respectively. In Fig. 2d–i, the main peak observed at ca. 55 ppm belongs to tetrahedral framework aluminum species (Al^IV^) in zeolite framework^20^. The small peak at ca. −20 ppm was assigned to spinning sideband^29^. The fresh Na-ZSM-5 revealed that the Al^IV^ framework concentration increased with increasing Al_2_O_3_ content during the catalyst synthesis (decreasing the SiO_2_/Al_2_O_3_ molar ratio). No peaks of penta-coordinated aluminum species (Al^V^) (ca. 30 ppm), or octahedral aluminum species (Al^VI^) (ca. 0 ppm)^20^ were observed on fresh synthesized zeolites. The spent samples retained the Al^IV^ framework structure and no new peaks, of Al^V^ and Al^VI^ species, were produced, however, the intensity of the Al^IV^ species in zeolite framework peak decreased compared to the fresh samples, and the concentration change was proportional to the SiO_2_/Al_2_O_3_ molar ratio of catalyst, this confirms that dealumination occurred during the butene cracking reaction on these catalysts.

^29^Si MAS NMR spectra present peaks at ca. −112, −102, and −93 ppm which were attributed to the Si(4Si,0Al) site, Si atoms without neighboring Al atom, Si(3Si,1Al) site, Si atoms with one neighboring Al atom, and Si(2Si,2Al) site, Si atoms with two neighboring Al atoms, respectively^30,31^. The spectra of synthesized Na-ZSM-5 zeolites in this research showed resonance signals corresponding to previous studies^30,31^ as shown in Fig. 2j–o, the intensities of each signal for all samples are presented in Supplement Table S3. Based on Fig. 2j–o and Supplement Table S3, the fresh synthesized Na-ZSM-5 zeolites displayed one main resonance signal at ca. − 102 ppm corresponding to the Si(3Si,1Al) site, and a low intensity peak at ca. − 113 ppm, attributed to the Si atoms without neighboring Al atom structure. The fresh sample was also presented a small shoulder band, typical of two Si atoms with two neighboring Al atoms at ca. − 94 ppm. Nevertheless, the reaction time (300 min), the signal patterns of spent catalysts were notoriously changed. The band intensity at ca. − 102 ppm reduced, whereas the Si(4Si,0Al) site signal (ca. − 113 ppm) abruptly increased compared to the fresh zeolite catalyst. No peaks for Si(1Si,3Al) or Si(0Si,4Al) sites were observed on fresh and spent Na-ZSM-5 catalysts.

### Catalyst performance test

**Figure 3:**
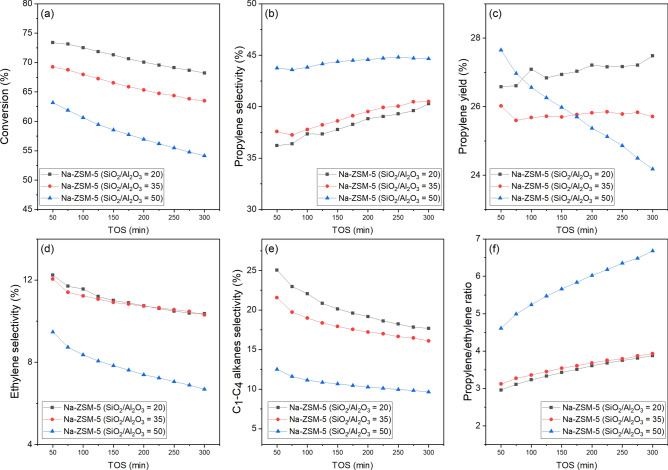
Performance of butene cracking reaction on Na-ZSM-5 catalysts with different SiO_2_/Al_2_O_3_ molar ratios at 500 °C under atmospheric pressure with WHSV = 3 h^−1^ , molar ratio of reactant between butene and N_2_ = 65:35, and 300 min of time on stream (TOS) (**a**) Conversion, (**b**) propylene selectivity, (**c**) propylene yield, (**d**) ethylene selectivity, (**e**) C_1_–C_4_ alkanes selectivity, and (**f**) Propylene/ethylene ratio. Note: the data from Supplement Table S4.

Figure 3 presents the performance of Na-ZSM-5 catalysts for butene cracking reaction as a function of time on stream (TOS). From Fig. 3a, the best stability for the butene cracking reaction was reached over the Na-ZSM-5 (SiO_2_/Al_2_O_3_ = 20), and the deactivation rate followed the trend of 7.03%, 8.31%, and 14.32% for SiO_2_/Al_2_O_3_ = 20, 35, and 50, respectively. This increase in deactivation led to a reduction in initial butene conversion rate. The propylene selectivity as a function of time enhanced with increasing the SiO_2_/Al_2_O_3_ molar ratio of Na-ZSM-5 catalysts as shown in Fig. 3b. Remarkably, the Na-ZSM-5 (SiO_2_/Al_2_O_3_ = 20) presented excellent propylene production with a final propylene yield of 27.48%, as displayed in Fig. 3c. The initial ethylene and light alkanes (C_1_–C_4_ alkanes) selectivities dropped when raising SiO_2_/Al_2_O_3_ molar ratio of Na-ZSM-5 catalysts, as illustrated in Fig. 3d,e, respectively.

### Coke characterization

**Figure 4:**
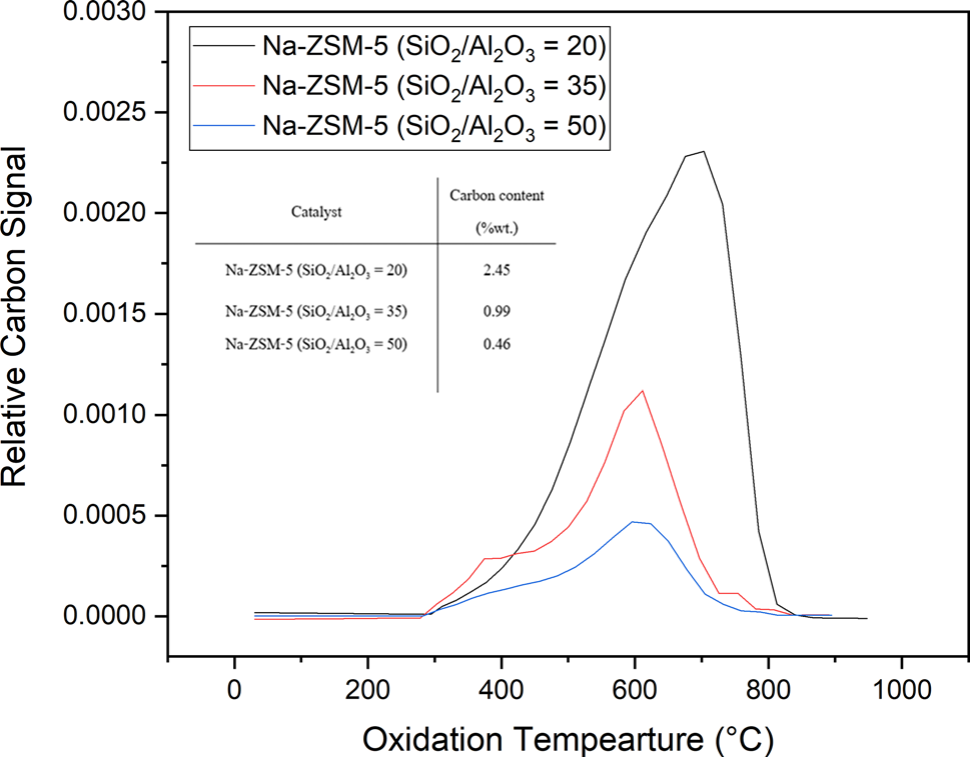
TPO profiles of spent Na-ZSM-5 catalyst with diffident SiO_2_/Al_2_O_3_ molar ratios after 300 min of TOS.

**Figure 5:**
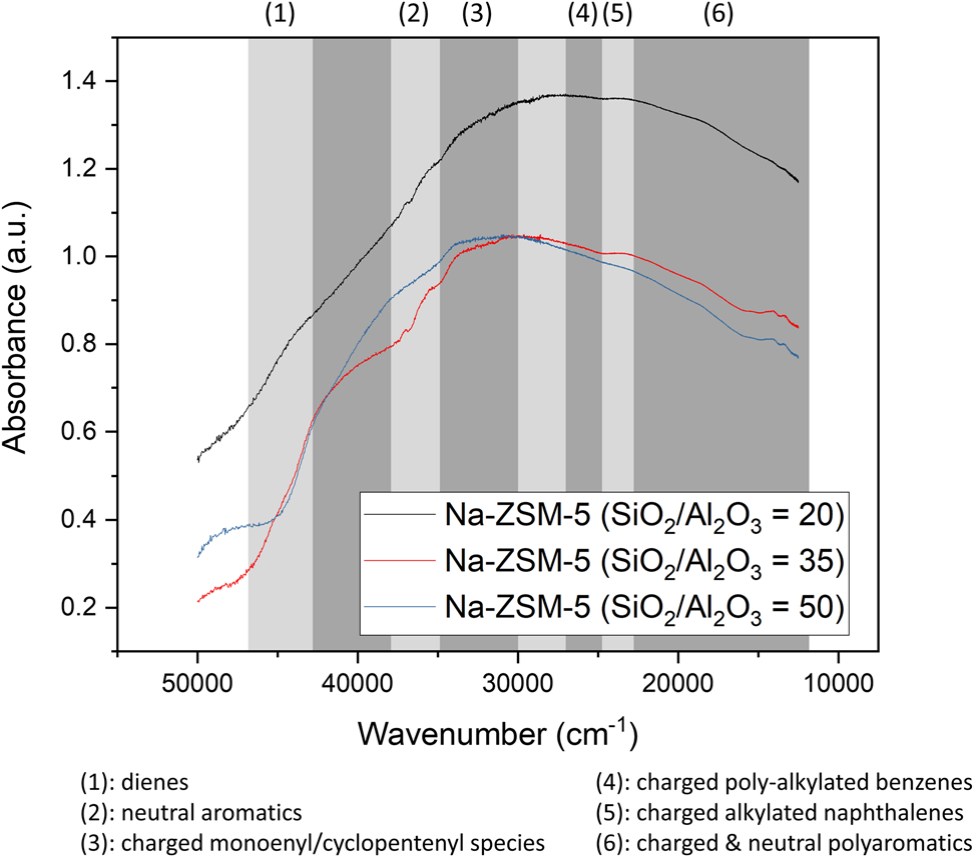
UV–Vis profiles of Na-ZSM-5 with diffident SiO_2_/Al_2_O_3_ molar ratios. Note: the organic species Refs.^14, 32–37^.

UV–Vis spectrometry and TPO were used to analyze the carbon species and weight percentage of carbon deposit over the Na-ZSM-5 surface after the butene cracking reaction, respectively. Figure 4 presents the profile and weight percentage of carbon content after the reaction of various catalysts. The main oxidation peaks of Na-ZSM-5 (SiO_2_/Al_2_O_3_ = 20, 35, 50) were appeared at 702 °C, 610 °C, and 604 °C, respectively (Fig. 4). The main band from TPO profiles shifted to higher temperatures when the SiO_2_/Al_2_O_3_ molar ratio of Na-ZSM-5 catalysts was reduced, and the coke content raised as illustrated in Fig. 4. The subtracted spectra between spent and fresh catalysts can provide information regarding coke species, formed during the reaction as depicted in Fig. 5. The spectra intensity of Na-ZSM-5 (SiO_2_/Al_2_O_3_ = 20) was the highest for all coke species, whereas the intensity bands corresponding to charged poly-alkylated benzenes, charged alkylated naphthalenes, and charged & neutral polyaromatics species of Na-ZSM-5 (SiO_2_/Al_2_O_3_ = 35) were higher than those of Na-ZSM-5 (SiO_2_/Al_2_O_3_ = 50).

## Discussion

It is widely accepted that the strong Brønsted acid site (Si–(OH)–Al) over acid-solid zeolite, especially for H-ZSM-5 catalysts, dominates the butene cracking reaction^6,38^. However, in the case that the catalyst lacks Si-(OH)-Al sites like Na-ZSM-5, the silanol group can act as an active site in this reaction and plays the same role as strong Brønsted acid site. The concentration of silanol group, determined by ^1^H MAS NMR technique as summarized in Supplement Table S3, correlated with the butene conversion over time, as depicted in Fig. 3a. Therefore, the silanol group on Na-ZSM-5 was the active site for butene cracking reaction, which confirmed previous findings where silanol groups could play the same role as the strong Brønsted acid site and these were active sites for different reactions such as 1-butene cracking reaction (silanol nest over silicalite-1)^39^, toluene methylation (silanol nest over silicalite-1)^22^, and methanol conversion (hydrogen-bonded silanol groups over methanol conversion)^40^.

In the case of C_4_ olefins cracking reaction, the mechanism is more complicated. The main reaction starts when the acid site of the catalyst is protonated, this proton reacts with C_4_^=^ species to form the carbenium ion (C_4_^+^). Then, the oligomerization will occur and crack to light alkenes in various pathways depending on acid strength of the catalyst^41^. Finally, the light alkanes and aromatics are generated via hydrogen transfer reaction^42^ and dehydrogenation–aromatization^43^ reactions, respectively. Both of these reactions require a strong acid site on catalyst^39^. For Na-ZSM-5 catalyst, the silanol groups worked as the active site in butene cracking reaction. The cracking reaction is composed of three main parallel reactions: (1) C_8_^I^ → 2C_4_^=^, (2) C_8_^II^ → C_2_^=^ + C_6_^=^, and (3) C_8_^III^ → C_3_^=^ + C_5_^=^. Stronger acid site favors the pathway (2) over pathway (3)^6,44,45^. The ratio of propylene to ethylene decreased with a lower SiO_2_/Al_2_O_3_ molar ratio of Na-ZSM-5 as displayed in Fig. 3f. Therefore, the lower SiO_2_/Al_2_O_3_ molar ratio of Na-ZSM-5 could generate stronger acid site, leading to higher selectivity of pathway (2) over pathway (3). Besides, when acid strength over the Na-ZSM-5 was stronger as presented in Fig. 1, the hydrogen transfer reaction to propylene to produce propane was improved, thus, the propylene selectivity of Na-ZSM-5 at low SiO_2_/Al_2_O_3_ molar ratio was lower than the Na-ZSM-5 with high SiO_2_/Al_2_O_3_ molar ratio. This result was confirmed by earlier research reported by Lin et al.^41^. They found that the acid strength distribution could control the butene conversion and the propylene/ethylene mole ratio (P/E ratio) and the P/E ratio increased with decreasing the density of strong acid site over the catalyst. The sodium-containing ZSM-5 catalyst retards the hydrogen transfer reaction, hindering the light alkanes (C_1_–C_4_ alkanes) production since the Na-ZSM-5 only had silanol groups in the zeolite framework and these acid sites were not strong enough to activate this side reaction. However, the hydrogen transfer reaction slightly occurred over silanol groups. The light alkanes production was associated with the acid strength of the Na-ZSM-5 catalysts and increased correspondingly as illustrated in Figs. 1, 3e, and reported in Supplement Table S2. Catalytic performance for butene cracking reaction of the Na-ZSM-5 (SiO_2_/Al_2_O_3_ = 20), and the ZSM-5 (H-ZSM-5 and silicalite-1) from other studies are compared in Table 1.

**Table 1:** The comparison of ZSM-5 (H-ZSM-5 and silicalite-1 catalysts) in butene cracking reaction.

Year	Catalyst name	Reaction condition	Butenes conversion (%)	Propylene yield (%)	References
2020	Na-ZSM-5 (SiO_2_/Al_2_O_3_ = 20)	Temperature = 500 °C, WHSV = 3 h^−1^ (C_4_H_8_:N_2_ = 65:35)	73.40^a^	26.58^a^	This study
2005	H-ZSM-5 (Si/Al_2_ = 50)	Temperature = 620 °C, WHSV = 3.5 h^−1^ (Pure 1-butene (> 99.5%))	~ 98^b^	~ 12^b^	^2^
2005	H-ZSM-5 (Si/Al_2_ = 50)	Temperature = 620 °C, WHSV = 3.5 h^−1^ (Pure 1-butene (> 99.5%))	~ 90^c^	~ 20^c^	^46^
2007	H-ZSM-5 (SiO_2_/Al_2_O_3_ = 40)	Temperature = 600 °C, WHSV = 3.2 h^−1^ (C_4_-olefin feed mixtures)	~ 84^d^	~ 23^d^	^6^
2009	H-ZSM-5 (Si/Al = 35)	Temperature = 530 °C, WHSV = 13 h^−1^ (C_4_H_8_:N_2_ = 3:1)	98.70^e^	2.10^e^	^47^
2011	H-ZSM-5 (SiO_2_/Al_2_O_3_ = 26)	Temperature = 510 °C, WHSV = 3.5 h^−1^ (C_4_H_8_:C_4_H_10_ = 0.48:0.52)	~ 98f.	~ 15f.	^48^
2014	H-ZSM-5 (Si/Al_2_ = 23)	Temperature = 500 °C GHSV = 900 h^−1^ (C_4_H_8_:N_2_ = 1:5)	99.90^ g^	0.40^ g^	^39^
2014	H-ZSM-5 (Si/Al_2_ = 80)	Temperature = 500 °C GHSV = 900 h^−1^ (C_4_H_8_:N_2_ = 1:5)	96.20^ g^	10.20^ g^	^39^
2014	H-ZSM-5 (Si/Al_2_ = 280)	Temperature = 500 °C GHSV = 900 h^−1^ (C_4_H_8_:N_2_ = 1:5)	88.80^ g^	20.25^ g^	^39^
2014	Silicalite-1	Temperature = 500 °C GHSV = 900 h^−1^ (C_4_H_8_:N_2_ = 1:5)	66.70^ g^	20.08^ g^	^39^
2015	H-ZSM-5 (Si/Al_2_ = 50)	Temperature = 550 °C, WHSV = 5 h^−1^ (Pure 1-butene (> 99.5%))	~ 90^ h^	~ 18^ h^	^49^
2017	H-ZSM-5 (SiO_2_/Al_2_O_3_ = 30)	Temperature = 500 °C, Space time = 0.32 g catalyst*h*(mol_C_) ^−1^ (90 vol% 1-butene in He)	~ 98^i^	~ 15^i^	^50^
2017	Steamed H-ZSM-5 at 500 °C (SiO_2_/Al_2_O_3_ = 30)	Temperature = 500 °C, Space time = 0.32 g catalyst*h*(mol_C_) ^−1^ (90 vol% 1-butene in He)	~ 90^i^	~ 25^i^	^50^

*Note: ^a^ The value at 50 min of TOS, ^b^ The value at 2 min of TOS, ^c^ The value at 1 h of TOS, ^d^ The initial value, ^e^ The value at 40 min of TOS, ^f^ The value at 8.5 h of TOS, ^g^ The value at 1 h of TOS, ^h^ The value at 2 h of TOS, and ^i^ The value at 0 min of TOS.

The H-ZSM-5^2^ and silicalite-1^39^ were previously reported to be suitable catalysts in the butene cracking reaction. H-ZSM-5 (SiO_2_/Al_2_O_3_ = 20) and Na-ZSM-5 (SiO_2_/Al_2_O_3_ = ∞, silicalite-1) catalysts were synthesized and examined in butene cracking reaction, the related information on catalytic performance and the characterization are provided in Supplement Figures S3 and S4, respectively. Based on Supplement Figure S3a, the H-ZSM-5 (SiO_2_/Al_2_O_3_ = 20) exhibited a higher butene conversion than Na-ZSM-5 (SiO_2_/Al_2_O_3_ = 20), because the H-ZSM-5 has strong Brønsted acid sites related to Si–(OH)–Al structure, which has a characteristic FT-IR signal at ca. 3,610 cm^−1^^25^ (Supplement Figure S3f). For the Na-ZSM-5 (SiO_2_/Al_2_O_3_ = ∞, silicalite-1) performance, the butene conversion (Supplement Figure S4a) significantly decreased since the intensity of the band assigned to silanol group (Supplement Figure S4f) over this catalyst was extremely low as compared to the Na-ZSM-5 catalysts. For the product distribution, the H-ZSM-5 (SiO_2_/Al_2_O_3_ = 20) granted higher light alkane (C_1_–C_4_) selectivity than Na-ZSM-5 (SiO_2_/Al_2_O_3_ = 20) (sSpplement Figure S3b) because the acid strength of H-ZSM-5 (SiO_2_/Al_2_O_3_ = 20) was stronger than Na-ZSM-5 (SiO_2_/Al_2_O_3_ = 20) (Supplement Figure S3e). Therefore, the hydrogen transfer reaction to produce light alkanes was promoted over H-ZSM-5 and hindered on Na-ZSM-5, leading to a lower propylene yield for H-ZSM-5 (SiO_2_/Al_2_O_3_ = 20) (Supplement Figure S3c). The propylene yield for Na-ZSM-5 (SiO_2_/Al_2_O_3_ = ∞) was low (Supplement Figure S4c) because of the lower butene conversion over this catalyst (Supplement Figure S4a). To evaluate the effect of Na and Al structures, the performance of Na-ZSM-5 (SiO_2_/Al_2_O_3_ = 20), H-ZSM-5 (SiO_2_/Al_2_O_3_ = 20), and Na-ZSM-5 (SiO_2_/Al_2_O_3_ = ∞, silicalite-1) in the butene cracking reaction were contrasted. The highest propylene yield and stability after 300 min of TOS were achieved by Na-ZSM-5 (SiO_2_/Al_2_O_3_ = 20).

In order to investigate the effect of WHSV in butene cracking reaction, Na-ZSM-5 (SiO_2_/Al_2_O_3_ = 20) was tested at different WHSVs 1.5 h^−1^, 3.0 h^−1^, and 6.0 h^−1^ (Supplement Figure S5). When increasing the WHSV, the butene conversion and ethylene selectivity decreased, and the propylene to ethylene ratio raised. These results corresponded with previous studies^41^. Decreasing the WHSV, lowered the propylene selectivity, and enhanced the light alkanes production. Consequently, hydrogen transfer reaction was inversely proportional to the WHSV. The WHSV = 3.0 h^−1^ was found to be the best condition providing the highest propylene yield at the initial TOS and the lowest deactivation rate after 300 min of TOS in butene cracking reaction.

**Figure 6:**
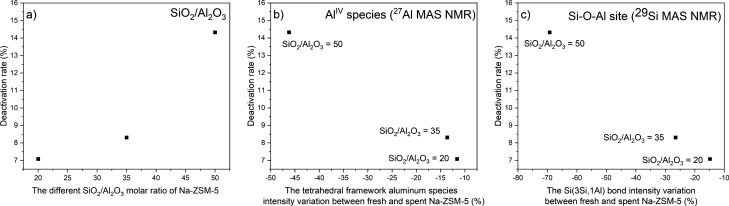
The correlation between Na-ZSM-5 catalyst deactivation rate and catalyst property results (**a**) the different SiO_2_/Al_2_O_3_ molar ratio of Na-ZSM-5, (**b**) the tetrahedral framework aluminum species intensity variation between fresh and spent Na-ZSM-5 (%), and (**c**) the Si(3Si,1Al) bond intensity variation between fresh and spent Na-ZSM-5 (%).

**Figure 7:**
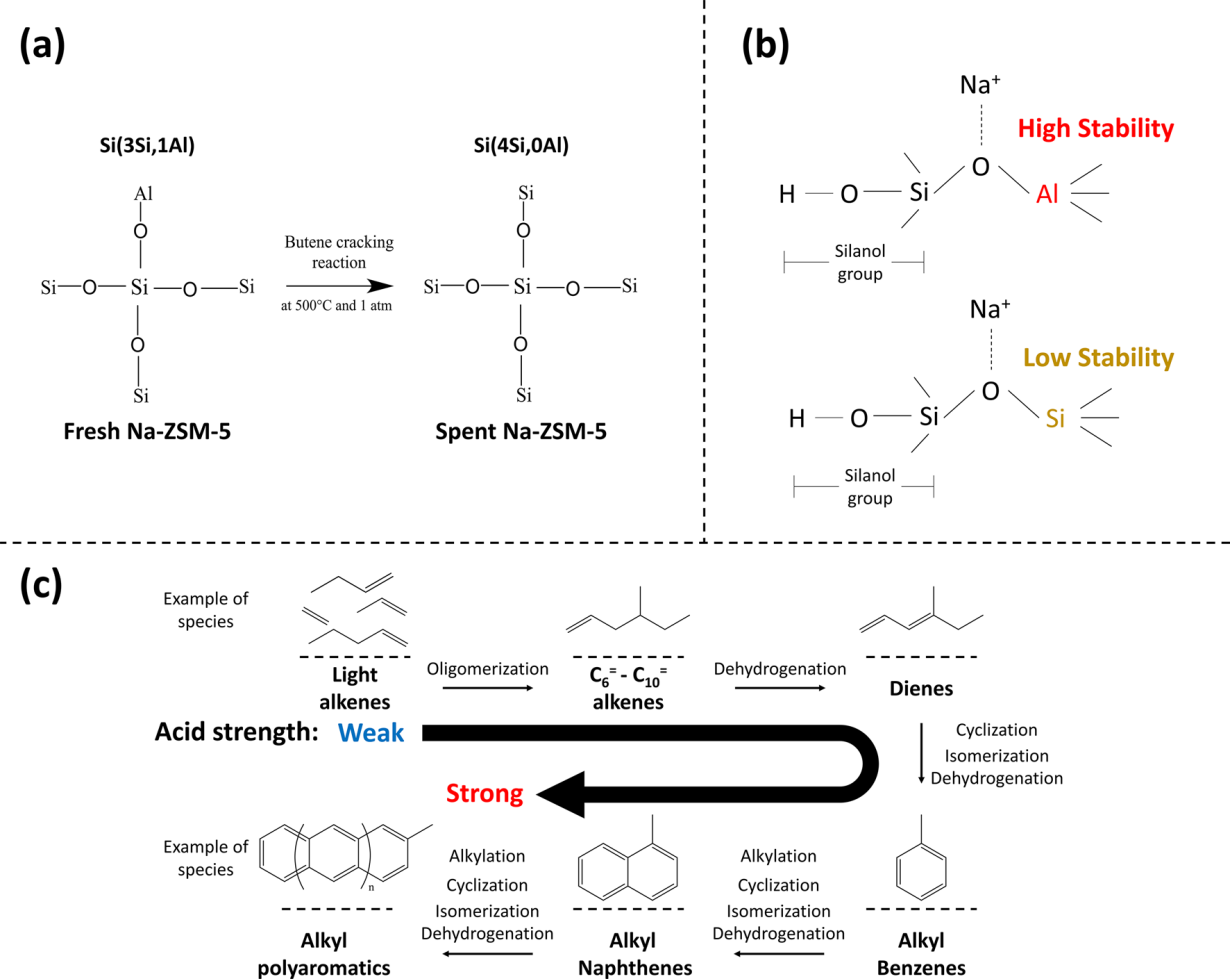
The proposed models (**a**) the proposed mechanism of the transformation of Si(3Si,1Al) to Si(4Si,0Al) site, (**b**) the proposed model of Si–ONa–Al and Si–ONa–Si bonds, and (**c**) the proposed coke formation mechanism.

The role of Al atom in zeolite structure was related to the Na-ZSM-5 catalysts deactivation as presented in the ^27^Al and ^29^Si MAS NMR results. The dealumination of tetrahedral framework aluminum species (Al^IV^) could occur during the butene reaction, and the loss of Al^IV^ species was followed with the ^29^Si MAS NMR spectra. As depicted in Fig. 6a, the Na-ZSM-5 catalyst deactivation rate, and the SiO_2_/Al_2_O_3_ molar ratio are directly related. The lower Al^IV^ species band intensity of spent Na-ZSM-5 catalyst, compared with fresh samples, confirmed the loss of Al^IV^ species in zeolite framework structure during the reaction, and the dealumination was remarkably escalated with lower aluminum content in zeolite structure, as displayed in Fig. 6b. These results were confirmed by Al-Khattaf et al.^51^ previous research, in which they found that the dealumination of H-ZSM-5 (SiO_2_/Al_2_O_3_ = 80) occurred because the ratio of Al^VI^ to Al^IV^ species increased after steaming from 0.009 (fresh) to 0.015 (steamed at 350 °C). The ^29^Si MAS NMR spectra of silicon environments are presented in Fig. 2j–o, a difference between fresh and spent samples is evident. The main type of silicon environment on fresh Na-ZSM-5 was Si(3Si,1Al) site, implying the presence of Si–O–Al bond in zeolite structure. While after the reaction, the Si(4Si,0Al) site was the major silicon surrounding type. This result confirms that Si–O–Al bond in the zeolite framework was broken during the cracking reaction, leading to dealumination. Based on Fig. 6c, when the SiO_2_/Al_2_O_3_ molar ratio raised, the Si–O–Al bond intensity variation between fresh and spent catalyst was higher, leading to an increase of deactivation rate. This outcome verified that the Si–O–Al bond was lost, and Si–O–Si bond formed during the reaction as illustrated in Fig. 7a. However, the framework of spent samples retained the typical MFI structure of ZSM-5 catalysts (supplement figure S1). To further investigate the effect of sodium bulk content (Supplement Table S2) on catalyst stability during the reaction, the sodium content over Na-ZSM-5 catalyst was measured. Sodium stabilizes the Si–O–Al bond which at the same time increases the stability and activity of silanol groups. While the Si–O–Si structure reduces the stability of silanol groups. Therefore, a model for Si–ONa–Al and Si–ONa–Si structures was proposed in Fig. 7b to describe the role of Al and Na in the enhancement of catalyst stability in term of the Si–OH, as the active site. The experimental results on Na-ZSM-5 deactivation with SiO_2_/Al_2_O_3_ = 20, 35, 50, and ∞ after 300 min of TOS, followed the trend 7.03%, 8.31%, 14.32%, and 48.96%, respectively, confirm the proposed model. This result corresponded with the previous research of E. Derouane et al.^52^, where the Si–O–Al bond, in zeolite structures, properties were studied by quantum mechanical calculation. They concluded that the stability of Si–O–Al bond depends on the electropositive character of the charge compensating cation. The substitution of protons by sodium ions as the charge compensating cation of zeolite framework increased the stability of Si–O–Al bond, because the sodium has a more electropositive character.

Another deactivation cause was the carbon deposition on the catalyst surface. It is well established that coke formation takes place on a Si–(OH)–Al structure (strong Brønsted acid site)^11^. In the case of catalysts which only had the silanol groups as an active site, the coke deposits could also occur on these sites. The coke content over the catalysts after the reaction was determined by TPO technique. The TPO results are displayed in Fig. 4. It exhibited direct correlation with the number of silanol groups detected by ^1^H MAS NMR (Supplement Table S3). In case of Na-ZSM-5, the acid strength was not high enough to produce a high concentration of polyaromatic coke species. As shown in Fig. 5, the intensity in the range of UV–Vis band representing charged & neutral polyaromatics was lower than the range of UV–Vis bands representing charged poly-alkylated benzenes and charged alkylated naphthalenes. Moreover, the result determined by TPO and UV–Vis techniques demonstrated that the Na-ZSM-5 (SiO_2_/Al_2_O_3_ = 20) could generate a greater deal of coke content than the other samples (Na-ZSM-5 at SiO_2_/Al_2_O_3_ = 35, and 50) as seen from higher absorption intensity in the whole wavenumber range of coke species bands as depicted in Fig. 5. Besides, the charged & neutral polyaromatic coke species were more abundant over the H-ZSM-5 (SiO_2_/Al_2_O_3_ = 20), because of the highest acid strength of this catalyst (Supplement Figure S3e,h). The proposed mechanism for coke generation^41,53,54^ related to the acid strength of the catalyst is illustrated in Fig. 7c.

Finally, the results suggested the deactivation of Na-ZSM-5 catalyst in butene cracking reaction was strongly affected by the removal of framework aluminum in zeolite structure, while the coke formation was of minor effect. The loss of tetrahedral framework aluminum species during the reaction was caused by the Si–O–Al bond breaking in Si(3Si,1Al) site to form Si–O–Si in Si(4Si,0Al) site, and Na content of the catalyst correlated with the Si–O–Al structure. Hence, the Si–O–Al bond in zeolite framework with the Na atom to form Si-ONa-Al structure was the determining factor to inhibit the dealumination process, and improved the stability of the silanol groups over Na-ZSM-5 catalysts in butene cracking reaction.

## Conclusion

The Na-ZSM-5 catalysts (SiO_2_/Al_2_O_3_ molar ratio = 20, 35, and 50) were synthesized and tested for butene cracking reaction to investigate the catalyst deactivation and coke formation. Based on the characterization, the fresh Na-ZSM-5 presented the typical MFI structure, and only silanol groups (Si–OH) were detected over Na-ZSM-5 catalyst surface as the active sites. The butene conversion was associated to the acid strength of these sites. The coke content exhibited a similar trend as the butene conversion. Regarding catalyst deactivation, the dealumination during the reaction critically affected the Na-ZSM-5 stability due to the loss of the tetrahedral framework aluminum species. Consequently, the break of Si–O–Al bonds in Si(OSi)_3_OAl framework led to the formation of new Si–O–Si bonds and Si(OSi)_4_ structures. Also, the degree of dealumination of Na-ZSM-5 catalyst was a function of the alumina content in zeolite structure (SiO_2_/Al_2_O_3_ molar ratio), and the relation between Si–O–Al bond stability and sodium content of catalyst was established. The formation of Si–ONa–Al structure led to enhanced silanol group stability. Finally, the Na-ZSM-5 (SiO_2_/Al_2_O_3_ = 20) was the most promising catalyst in this study rendering the highest propylene yield, catalytic activity, and stability.

## Method

### Material synthesis

The Na-ZSM-5 zeolite catalyst was synthesized by rapid crystallization procedure^55^ as reported by the previous study^17^. Sodium silicate solution (Merck), and aluminum sulfate octadecahydrate (UNIVAR) were used as the silica source, and alumina source, respectively. The template was tetrapropylammonium bromide (Sigma-Aldrich), and the alkali source was sodium hydroxide (Merck). The molar composition of the resulting synthesis mixture gel with different SiO_2_/Al_2_O_3_ molar ratio was 0.85Na_2_O:1SiO_2_:aAl_2_O_3_:0.24TPABr:118.64H_2_O, where a = 0.05 (SiO_2_/Al_2_O_3_ = 20), 0.03 (SiO_2_/Al_2_O_3_ = 35), and 0.02 (SiO_2_/Al_2_O_3_ = 50), respectively. Then, the gel was mixed together, and moved to a stainless-steel autoclave with 2 steps of hydrothermal process, the target temperature of steps 1 and 2 were 160 °C (60 °C/h, and 2 h of crystallization time), and 210 °C (10 °C/h, and 5 h of crystallization time), respectively. Afterward, the observed crystal was rinsed with deionized water until pH = 7.0, and dried at 110 °C in oven overnight. Subsequently, the calcination process with air stream was operated at 550 °C for 3.5 h. Finally, the Na-ZSM-5 catalysts (SiO_2_/Al_2_O_3_ = 20, 35, and 50) were collected, respectively.

The Na-ZSM-5 (SiO_2_/Al_2_O_3_ = ∞, silicalite-1) and H-ZSM-5 (SiO_2_/Al_2_O_3_ = 20) were synthesized and the related information on catalytic performance and the characterization were provided in Supplementary information. The Na-ZSM-5 without the alumina source (SiO_2_/Al_2_O_3_ = ∞) was synthesized with the same method which was mentioned earlier and the H-ZSM-5 (SiO_2_/Al_2_O_3_ = 20) was further synthesized from Na-ZSM-5 (SiO_2_/Al_2_O_3_ = 20). The ion-exchange with 1 M ammonium nitrate solution (NH_4_NO_3_) at 80 °C for 2 h was applied to transfer Na^+^ ion to NH_4_^+^ ion in ZSM-5 structure, and then the catalyst was rinsed with deionized water, dried in the oven, and calcined as same as the method of Na-ZSM-5 which was mentioned earlier, respectively. Finally, the H-ZSM-5 with 20 of the SiO_2_/Al_2_O_3_ molar ratio was observed.

### Catalyst characterization

The crystallinity and crystal structure of fresh and spent samples were analyzed by X-ray powder diffraction (XRD) technique with a Bruker AXS D8 Advance (Ni-filtered CuK_α_ radiation). The 2 range between 5° and 50° with a step size of 0.01 was applied to record in XRD pattern of the sample. The X-ray fluorescence (XRF) with a Bruker S8 TIGER was used to analyze the bulk composition of catalyst sample. The acidity strength was characterized by temperature-programmed desorption of ammonia (NH_3_-TPD) technique with a Micromeritics Chemisorb 2,750 automated system. The sample weight (0.1 g) was moved into the U-quartz tube reactor under 25 ml/min of He gas stream at 550 °C in a preheating step, then the temperature was dropped to 40 °C to adsorb the ammonia under 25 ml/min of 15% NH_3_/He mixed gas stream for half an hour. Subsequently, the physisorbed ammonia was eliminated with He gas stream. Finally, the desorbed ammonia was recorded by TCD detector with linearly temperature increasing from 40 to 600 °C (10 °C/min). The stretching vibration of OH groups on catalyst sample was determined by the Fourier Transform-Infrared Spectroscopy (FT-IR) with a Bruker VERTEX 70v FT-IR Spectrometer. The 0.2 g of sample was transferred into the in situ IR cell with the KBr windows to preheat at 550 °C (10 °C/min) for 1 h with 25 ml/min of N_2_ gas stream. Afterward, the temperature was reduced to 40 °C for the OH group spectra recording, subtracting automatically with the background spectrum. The Fourier Transform Nuclear Magnetic Resonance Spectrometer 400 MHz (Solid) with a Bruker AVANCE III HD (Ascend 400 WB) spectrometer (4 mm diameter rotor, 8 kHz of speed rate, and 400.20 MHz of resonance frequency) was used to characterize the acidity structure, type of aluminum, and Si environment by ^1^H, ^27^Al, and ^29^Si MAS NMR methods over the sample, respectively. The coke content and species were identified by the temperature-programmed oxidation (TPO) and ultraviolet–visible spectroscopy (UV–Vis) techniques, respectively. The TPO profile was analyzed by a Micromeritics Chemisorb 2,750 automated system and the CO_2_ formation was detected by a gas chromatography with Rt-Q-BOND—Fused Silica Plot column. The 0.05 g of spent sample was weight and transferred into the U-quartz tube reactor under 25 ml/min of 1% O_2_/He mixed gas stream and the signal was recorded with 5 °C/min of step size in the range between room temperature and 970 °C. The Lambda 650 UV–Vis spectrophotometer was applied to characterize the intensity signal variation between spent and fresh catalyst samples at the range from 12,500 to 50,000 cm^−1^.

### Catalyst testing

The fixed-bed tubular reactor with a K type of thermocouple was used to test the catalytic cracking reaction of butene. The reactor was made up from stainless steel with 19.05 mm of inner reactor diameter. The steps of reaction testing under atmospheric pressure were following: (1) the catalyst sample (1 g) was preheated at 550 °C with N_2_ for 1 h, and (2) the reaction temperature was at 500 °C with the weight hourly space velocity (WHSV) of the reactant gas at 3 h^−1^ (the molar composition reactant gas between butene and N_2_ was 65:35). The product compositions were evaluated by a gas chromatography, an Agilent J&W HP-PLOT Al_2_O_3_ S with FID detector. The conversion, product selectivity, product yield, and deactivation rate after 300 min with time on stream were determined by Eqs. (1) – (4), respectively.

1 $${\text{Conversion}}\left( {\text{X}} \right) = \frac{{W_{0} - W_{t} }}{{W_{0} }} \times 100\%$$

2 $${\text{Product}}\;{\text{selectivity}} = \frac{{(W_{i} )_{t} }}{{W_{0} - W_{t} }} \times 100\% ,$$

3 $${\text{Product}}\;{\text{yield}} = Conversion_{t} \times Product \, selectivity_{t} ,$$

4 $${\text{Deactivation}}\;{\text{rate}} = \frac{{X_{0} - X_{f} }}{{X_{0} }} \times 100\% ,$$

Here W_0_, W_t_, and (W_i_)_t_ are defined as the weight percentages of butenes in the feed, of butenes in the product, and of any hydrocarbons in the product, detected by the gas chromatography, respectively.

And X_0_ and X_f_ are defined as the butene conversion at 50 min after reaction test and 300 min after reaction test, respectively.

## Supplementary information

Supplementary Information.
