# Supplementary material for: Role of Al in Na-ZSM-5 zeolite structure on catalyst stability in butene cracking reaction
Source: Sci Rep. 2020 Aug 12;10:13643. doi: 10.1038/s41598-020-70568-z (PMC7424521; doi:10.1038/s41598-020-70568-z)
Supplement: Supplementary file 1 — Supplementary Information. [file 41598_2020_70568_MOESM1_ESM.docx]

**SUPPLEMENTARY INFORMATION**

**Role of Al in zeolite structure on catalyst stability in butene reaction**

Chanon Auepattana-aumrung^a^, Victor Márquez^a^, Sippakorn Wannakao^b^, Bunjerd Jongsomjit^a^, Joongjai Panpranot^a^ , and Piyasan Praserthdam^a,*^

^a^ Center of Excellence on Catalysis and Catalytic Reaction Engineering, Department of Chemical Engineering, Faculty of Engineering, Chulalongkorn University, Bangkok 10330, Thailand

^b^ SCG Chemicals, Co., Ltd., 1 Siam Cement Road, Bangsue, Bangkok 10800, Thailand

Corresponding authors: piyasan.p@chula.ac.th (P. Praserthdam).

**SUPPLEMENTARY INFORMATION**

Supplementary Figure 1: XRD patterns of fresh and spent Na-ZSM-5 with different SiO_2_/Al_2_O_3_ molar ratio (a) Na-ZSM-5 (SiO_2_/Al_2_O_3_ molar ratio = 20), (b) Na-ZSM-5 (SiO_2_/Al_2_O_3_ molar ratio = 35), and (a) Na-ZSM-5 (SiO_2_/Al_2_O_3_ molar ratio = 50)

Supplementary Figure 2: FT-IR spectra of OH groups on Na-ZSM-5 with different SiO_2_/Al_2_O_3_ molar ratio

Supplementary Figure 3: The related information on catalytic performance and the characterization for H-ZSM-5 (SiO_2_/Al_2_O_3_ = 20) in butene cracking reaction (a) Conversion, (b) Propylene selectivity, (c) Propylene yield, (d) XRD of fresh and spent catalysts, (e) NH_3_-TPD profile, (f) FT-IR spectra of OH groups, (g) TPO profile, and (h) UV-VIS profile

Supplementary Figure 4: The related information on catalytic performance and the characterization for Na-ZSM-5 (SiO_2_/Al_2_O_3_ = ∞) in butene cracking reaction (a) Conversion, (b) Propylene selectivity, (c) Propylene yield, (d) XRD of fresh and spent catalysts, (e) NH_3_-TPD profile, (f) FT-IR spectra of OH groups, (g) TPO profile, and (h) UV-VIS profile

Supplementary Figure 5: Performance of butene cracking reaction on Na-ZSM-5 (SiO_2_/Al_2_O_3_ = 20) with different WHSV at 500 °C under atmospheric pressure, molar ratio of reactant between butene and N_2_ = 65:35, and 300 min of time on stream (TOS) (a) Conversion, (b) Propylene selectivity, (c) Propylene yield, (d) Ethylene selectivity, (e) C_1_-C_4_ alkanes selectivity, and (f) Propylene/ethylene ratio

Note: the data from supplement table S4

Supplementary Table 1: The amount of sodium content in the bulk of Na-ZSM-5 catalyst

| Catalyst | Na (%wt.)^a^ | Al (%wt.)^b^ |
| --- | --- | --- |
| Na-ZSM-5 (SiO_2_/Al_2_O_3_ = 20) | 1.70 | 2.39 |
| Na-ZSM-5 (SiO_2_/Al_2_O_3_ = 35) | 1.24 | 1.47 |
| Na-ZSM-5 (SiO_2_/Al_2_O_3_ = 50) | 1.07 | 1.17 |
| Na-ZSM-5 (SiO_2_/Al_2_O_3_ = ∞) | 0.68 | - |

^a^ The Na element content by XRF analysis

^b^ The Al element content by XRF analysis

Supplementary Table 2: The number of ZSM-5 acidity with different SiO_2_/Al_2_O_3_ molar ratio

| Catalyst | The number of acidity (mmol/g catalyst) | | | |
| --- | --- | --- | --- | --- |
|  | Weak acid | Medium acid | Strong acid | Total acid |
| Na-ZSM-5 (SiO_2_/Al_2_O_3_ = 20) | 46.20 | 43.70 | 1.00 | 90.90 |
| Na-ZSM-5 (SiO_2_/Al_2_O_3_ = 35) | 44.80 | 32.00 | 0.00 | 76.90 |
| Na-ZSM-5 (SiO_2_/Al_2_O_3_ = 50) | 40.70 | 20.30 | 0.00 | 61.00 |
| Na-ZSM-5 (SiO_2_/Al_2_O_3_ = ∞) | 2.60 | 1.30 | 0.00 | 3.90 |
| H-ZSM-5 (SiO_2_/Al_2_O_3_ = 20) | 31.50 | 40.70 | 27.20 | 99.40 |

Supplementary Table 3: The ^1^H, ^27^Al, and ^29^Si MAS NMR result of the samples

| Catalyst | The intensity of relative peak area (a.u.) | | | | |
| --- | --- | --- | --- | --- | --- |
|  | ^1^H MAS NMR | ^27^Al MAS NMR | ^29^Si MAS NMR | | |
|  | Si-OH | Al^IV^ | Si  (4Si,0Al) | Si  (3Si,1Al) | Si  (2Si,2Al) |
| Na-ZSM-5 (SiO_2_/Al_2_O_3_ = 20) Fresh | 7.62*10^8^ | 4.80*10^9^ | 3.45*10^8^ | 1.59*10^9^ | 5.94*10^7^ |
| Na-ZSM-5 (SiO_2_/Al_2_O_3_ = 35) Fresh | 2.72*10^8^ | 4.35*10^9^ | 2.84*10^8^ | 1.48*10^9^ | 1.84*10^8^ |
| Na-ZSM-5 (SiO_2_/Al_2_O_3_ = 50) Fresh | 2.05*10^8^ | 4.34*10^9^ | 2.39*10^8^ | 1.47*10^9^ | 4.46*10^8^ |
| Na-ZSM-5 (SiO_2_/Al_2_O_3_ = 20) Spent | - | 4.24*10^9^ | 3.00*10^9^ | 1.35*10^9^ | 1.56*10^8^ |
| Na-ZSM-5 (SiO_2_/Al_2_O_3_ = 35) Spent | - | 3.76*10^9^ | 2.22*10^9^ | 1.09*10^9^ | 1.82*10^8^ |
| Na-ZSM-5 (SiO_2_/Al_2_O_3_ = 50) Spent | - | 2.34*10^9^ | 1.45*10^9^ | 4.51*10^8^ | 9.20*10^7^ |

Supplementary Table 4: The product distribution of ZSM-5 with different SiO_2_/Al_2_O_3_ molar ratio

| Na-ZSM-5 with SiO_2_/Al_2_O_3_ molar ratio = 20 | | | | | | | | | | | |
| --- | --- | --- | --- | --- | --- | --- | --- | --- | --- | --- | --- |
| TOS (min) | 50 | 75 | 100 | 125 | 150 | 175 | 200 | 225 | 250 | 275 | 300 |
| conversion | 73.40 | 73.14 | 72.53 | 71.87 | 71.30 | 70.63 | 70.07 | 69.57 | 69.13 | 68.70 | 68.24 |
| C_1_-C_4_ alkanes | 25.05 | 22.97 | 22.07 | 20.85 | 20.14 | 19.6 | 19.18 | 18.63 | 18.24 | 17.83 | 17.69 |
| ethylene | 12.24 | 11.70 | 11.56 | 11.20 | 11.01 | 10.89 | 10.75 | 10.61 | 10.49 | 10.39 | 10.37 |
| propylene | 36.21 | 36.39 | 37.35 | 37.34 | 37.78 | 38.27 | 38.83 | 39.04 | 39.30 | 39.61 | 40.27 |
| C_5_^+^ | 26.49 | 28.94 | 29.01 | 30.62 | 31.08 | 31.24 | 31.25 | 31.72 | 31.96 | 32.16 | 31.69 |
| Na-ZSM-5 with SiO_2_/Al_2_O_3_ molar ratio = 35 | | | | | | | | | | | |
| TOS (min) | 50 | 75 | 100 | 125 | 150 | 175 | 200 | 225 | 250 | 275 | 300 |
| conversion | 69.24 | 68.76 | 67.97 | 67.27 | 66.56 | 65.88 | 65.34 | 64.73 | 64.38 | 63.82 | 63.49 |
| C_1_-C_4_ alkanes | 21.58 | 19.74 | 19.00 | 18.37 | 17.92 | 17.55 | 17.23 | 17.01 | 16.65 | 16.46 | 16.11 |
| ethylene | 12.05 | 11.40 | 11.23 | 11.07 | 10.91 | 10.83 | 10.73 | 10.65 | 10.56 | 10.47 | 10.31 |
| propylene | 37.58 | 37.24 | 37.78 | 38.23 | 38.61 | 39.11 | 39.52 | 39.93 | 40.04 | 40.47 | 40.49 |
| C_5_^+^ | 28.79 | 31.62 | 31.99 | 32.33 | 32.56 | 32.50 | 32.52 | 32.42 | 32.74 | 32.59 | 33.09 |
| Na-ZSM-5 with SiO_2_/Al_2_O_3_ molar ratio = 50 | | | | | | | | | | | |
| TOS (min) | 50 | 75 | 100 | 125 | 150 | 175 | 200 | 225 | 250 | 275 | 300 |
| conversion | 63.20 | 61.87 | 60.61 | 59.44 | 58.52 | 57.75 | 56.94 | 56.20 | 55.48 | 54.78 | 54.15 |
| C_1_-C_4_ alkanes | 12.52 | 11.60 | 11.14 | 10.88 | 10.67 | 10.46 | 10.27 | 10.11 | 9.96 | 9.83 | 9.67 |
| ethylene | 9.48 | 8.74 | 8.36 | 8.07 | 7.84 | 7.62 | 7.40 | 7.24 | 7.06 | 6.90 | 6.69 |
| propylene | 43.75 | 43.59 | 43.82 | 44.17 | 44.39 | 44.5 | 44.56 | 44.71 | 44.81 | 44.72 | 44.66 |
| C_5_^+^ | 34.24 | 36.07 | 36.68 | 36.89 | 37.11 | 37.41 | 37.77 | 37.93 | 38.17 | 38.55 | 38.98 |
| Na-ZSM-5 with SiO_2_/Al_2_O_3_ molar ratio = ∞ | | | | | | | | | | | |
| TOS (min) | 50 | 75 | 100 | 125 | 150 | 175 | 200 | 225 | 250 | 275 | 300 |
| conversion | 16.4 | 14.67 | 13.34 | 12.35 | 11.53 | 10.81 | 10.24 | 9.67 | 9.22 | 8.79 | 8.37 |
| C_1_-C_4_ alkanes | 10.93 | 10.91 | 11.04 | 11.23 | 11.42 | 11.65 | 11.86 | 12.12 | 12.33 | 12.58 | 12.87 |
| ethylene | 4.84 | 4.47 | 4.23 | 4.05 | 3.9 | 3.78 | 3.67 | 3.59 | 3.5 | 3.43 | 3.37 |
| propylene | 44.38 | 43.71 | 43.23 | 42.9 | 42.49 | 42.3 | 41.95 | 41.73 | 41.37 | 41.16 | 41.05 |
| C_5_^+^ | 39.86 | 40.91 | 41.5 | 41.82 | 42.21 | 42.26 | 42.52 | 42.56 | 42.8 | 42.83 | 42.72 |
| H-ZSM-5 with SiO_2_/Al_2_O_3_ molar ratio = 20 | | | | | | | | | | | |
| TOS (min) | 50 | 75 | 100 | 125 | 150 | 175 | 200 | 225 | 250 | 275 | 300 |
| conversion | 86.64 | 83.17 | 80.45 | 78.23 | 70.5 | 68.12 | 66.03 | 64.3 | 62.68 | 61.01 | 59.49 |
| C_1_-C_4_ alkanes | 46.1 | 33.5 | 27.3 | 23.26 | 22.1 | 19.99 | 18.54 | 16.81 | 15.56 | 14.48 | 13.61 |
| ethylene | 10.61 | 11 | 11.02 | 10.82 | 11.38 | 11.19 | 11.01 | 10.48 | 10.04 | 9.52 | 9.17 |
| propylene | 22.48 | 26.8 | 29.67 | 31.75 | 36.2 | 38.18 | 40.25 | 40.77 | 41.51 | 42.04 | 42.49 |
| C_5_^+^ | 20.82 | 28.7 | 32.01 | 34.16 | 30.32 | 30.63 | 30.19 | 31.93 | 32.9 | 33.95 | 34.72 |
| Na-ZSM-5 with SiO_2_/Al_2_O_3_ molar ratio = 20 (WHSV = 1.5 h^-1^) | | | | | | | | | | | |
| TOS (min) | 50 | 75 | 100 | 125 | 150 | 175 | 200 | 225 | 250 | 275 | 300 |
| conversion | 86.41 | 84.25 | 83.04 | 81.21 | 79.76 | 78.60 | 77.48 | 76.46 | 75.53 | 74.88 | 73.92 |
| C_1_-C_4_ alkanes | 49.15 | 40.91 | 35.84 | 32.1 | 29.26 | 26.95 | 25.04 | 23.55 | 22.29 | 21.01 | 20.41 |
| ethylene | 12.41 | 12.63 | 12.8 | 12.79 | 12.69 | 12.49 | 12.29 | 12.09 | 11.85 | 11.52 | 11.43 |
| propylene | 22.83 | 26.15 | 28.2 | 30.68 | 32.46 | 33.94 | 35.15 | 36.34 | 37.34 | 37.8 | 38.96 |
| C_5_^+^ | 15.61 | 20.31 | 23.16 | 24.42 | 25.59 | 26.61 | 27.52 | 28.02 | 28.53 | 29.67 | 29.2 |
| Na-ZSM-5 with SiO_2_/Al_2_O_3_ molar ratio = 20 (WHSV = 6.0 h^-1^) | | | | | | | | | | | |
| TOS (min) | 50 | 75 | 100 | 125 | 150 | 175 | 200 | 225 | 250 | 275 | 300 |
| conversion | 62.24 | 56.95 | 54.44 | 52.03 | 49.95 | 48.20 | 46.42 | 44.92 | 43.62 | 42.34 | 41.30 |
| C_1_-C_4_ alkanes | 22.6 | 18.81 | 16.57 | 14.86 | 13.75 | 12.94 | 12.36 | 11.87 | 11.47 | 11.15 | 10.84 |
| ethylene | 11.61 | 11.01 | 10.1 | 9.45 | 8.91 | 8.5 | 8.14 | 7.8 | 7.5 | 7.22 | 6.98 |
| propylene | 38.89 | 41.74 | 42.11 | 42.6 | 43.11 | 43.67 | 44.21 | 44.49 | 44.76 | 44.97 | 45.06 |
| C_5_^+^ | 26.91 | 28.45 | 31.2 | 33.1 | 34.24 | 34.9 | 35.28 | 35.84 | 36.26 | 36.66 | 37.13 |
